# Predictors of 1-year mortality in adult lung transplant recipients: a systematic review and meta-analysis

**DOI:** 10.1186/s13643-019-1049-x

**Published:** 2019-06-03

**Authors:** Farid Foroutan, Gordon Guyatt, Erik Friesen, Luis Enrique Colunga Lozano, Aman Sidhu, Maureen Meade

**Affiliations:** 1 Department of Multi-Organ Transplant, University Health Network, 200 Elizabeth St, Toronto, ON M5G 2C4 Canada; 2 Department of Health Research Methods, Evidence, and Impact, McMaster University, Hamilton, ON Canada; 3 University of Toronto, Toronto, ON Canada

**Keywords:** Lung transplantation, Systematic review, Meta-analysis, Prognostication

## Abstract

**Background:**

Upon surviving the first year post-lung transplantation, recipients can expect a median survival of 8 years. Within the first year, graft failure and multi-organ failure (possibly secondary to graft failure) are common causes of mortality. To better understand the prognosis within the first year, we plan on conducting a systematic review and meta-analysis of observational studies addressing the association between the patient, donor, and transplant operative factors and graft loss 1-year post-lung transplant.

**Methods:**

We searched MEDLINE, Embase, Cochrane Database of Systematic Reviews, Cochrane Central Register, and PubMed supplemental for non-MEDLINE records for observational studies identifying independent risk factors for early mortality (1 year) in adult lung transplant recipients. We plan on including cohort studies and secondary analyses of randomized controlled trials studying adult lung transplant recipients undergoing their first lung transplant, without any simultaneous organ transplant. We will conduct a random-effects meta-analysis that pools the effect estimates from all eligible studies to obtain a summary estimate and confidence interval for all independent non-therapeutic factors identified in the primary studies.

**Discussion:**

The results from this study may inform future guidelines on the selection of candidates and donors for transplantation and predictive model development and inform the decision-making process that the physician and patient undertake together. Furthermore, through the conduction of this review, we can identify the limitations with the current best evidence, which will encourage the need for studies with a better methodology to reassess the predictors of mortality.

**Electronic supplementary material:**

The online version of this article (10.1186/s13643-019-1049-x) contains supplementary material, which is available to authorized users.

## Background

Adult lung transplant recipients can expect a median survival of 8 years, conditional on surviving the first year post transplant [1]. Graft failure and multi-organ failure (possibly secondary to graft failure), however, are common causes of mortality within the first year, decreasing the conditional median survival from 8 to 5 years [1].

To better understand the risk of mortality within the first year post transplantation, the International Society of Heart and Lung Transplantation (ISHLT) identified the risk factors for 1-year mortality through the use of multivariable analysis [1]. Due to the lack of mandatory reporting to the ISHLT registry, however, there may be studies from centers assessing the same set of risk factors but not reporting their findings to the registry. Based on their unique cohort, the risk estimates and 95% confidence interval calculated may vary compared to that reported by the registry. The risk estimates obtained from individual studies may also vary from one another which further necessitate the need for one true overall effect estimate with a concomitant exploration of potential reasons for the observed inconsistency. For example, Borro et al. analyzed data from Spain and suggested an 8% relative reduction in hazard of mortality in patients undergoing double lung transplant compared to single lung transplant [2]. This association may have been observed by chance alone (95% CI crossing the boundary of no effect). Jacques et al. similarly analyzed Canadian data to suggest a 66% relative increase in the hazard of mortality for patients undergoing bilateral lung transplantation (an association that again could be by chance alone) [3]. Finally, Neurohr et al. analyzed the same predictor (bilateral lung transplantation) to observe a 66% relative decrease in hazard of mortality (95% CI 0.14 to 0.79) [4]. A non-systematic narrative review and/or registry study suffers by only highlighting the one statistically significant risk estimate obtained from a select sample of patients, and thus potentially ignoring the variability that may exist across a different population.

A systematic review and meta-analysis addressing the factors associated with mortality, identified by all individual studies, may better inform the relative independent effect of each risk factor in the context of patients suitable for transplantation. Among its advantages, by conducting a systematic review and meta-analysis, we may potentially generate hypotheses for sources of variations across studies assessing the risk factors for early mortality. We therefore plan on conducting a systematic review and meta-analysis of observational studies addressing the association between the patient, donor, and transplant operative factors and graft loss 1-year post-lung transplant.

## Research question

In adult (≥ 18 years) lung transplant recipients, what are the independent predictors of mortality at 1-year post-transplant?

## Objective

The objectives of this systematic review are as follows:

- To summarize all factors associated with mortality within the first year post-lung transplant
- To pool similar studies to obtain an overall point estimate and confidence interval of commonly identified risk factors for mortality at 1-year post transplant.

## Methods

We submitted our study protocol to the PROSPERO registry (submission ID: 132698). This study will systematically review observational studies identifying the independent risk factors for early mortality (1 year) in adult lung transplant recipients. We will conduct a meta-analysis that pools the effect estimates from all eligible studies to obtain a summary estimate and confidence interval for all independent factors identified in the primary studies.

### Criteria for study selection

The following are the criteria for the study selection:

1. Types of studies
  Observational studies (retrospective or prospective) and secondary analyses of randomized control trials.
2. Types of participants
  Adult (≥ 18 years) lung transplant recipients.
3. Type of exposure
  Any non-therapeutic independent predictor of mortality at 1-year post transplant related to the recipient, donor, and the transplant operation.
4. Types of outcome measures
  The primary outcome measure is 1-year mortality post transplant. Effect measures may be a relative risk, odds ratio, or hazard ratio.
5. Language
  All languages.

### Selection process

We will include observational studies and secondary analyses of randomized control trials that enrolled adult (at least 95% of population = 18 years) de novo transplant recipients, evaluating any factor associated with mortality using multivariable analysis (Cox proportional hazards models, logistic regression models), reporting more than 20 events. We will exclude studies assessing mortality beyond the first year if they do not use a time-to-event analysis that meets the proportional hazard assumption. We do not plan on excluding studies based on the language of publication.

We will exclude studies with insufficient information to generate estimates of effect for any predictor (lack of effect estimate, 95% confidence interval, *p* value to be combined in the meta-analysis). We will exclude duplicate studies assessing the same population without additional data on new predictors. If two studies assessed the same population and predictors, we will include the study with a larger sample size. If two studies assessed the same population, but the smaller study reports on a unique predictor, the larger study will inform other predictors whereas the small study will inform the unique predictor.

Due to mandatory reporting to the United Network of Organ Sharing (UNOS) registry, we will consider the risk estimates from this registry to represent all individual centers from the United States of America (USA) (thus excluding all individual studies from the USA for the same risk factor and time frame). Due to the lack of mandatory reporting to the ISHLT registry, however, we will exclude all studies querying this registry. We will trust our search strategy and screening process to capture patients included in the ISHLT registry.

Using standardized study eligibility forms (Additional file 2), paired reviewers will independently screen the titles and abstracts of identified citations and evaluate the full text of articles deemed potentially eligible by either reviewer. We will resolve disagreements in full-text screening through discussion or, if necessary, through adjudication by a third reviewer.

All citations will be imported into Covidence (Thomson Reuters) [5]. Ten reviewers (FF, EF, RZ, RK, AS, TW, AT, KM, SA, EA) will screen the titles and abstracts, as well as the full-text citations, independently and in duplicate. If a decision cannot be made on whether an article is eligible, reviewers will consult an independent adjudicator (GG).

### Study sources

We completed the search using searched MEDLINE, Embase, Cochrane Database of Systematic Reviews, Cochrane Central Register, and PubMed supplemental for non-MEDLINE records. We developed a broad strategy for predictors/prognosis as many of the types of articles that might meet the inclusion criteria would not necessarily be found by a more focused search on predictors or the types of statistical tools used in the studies. That filter consists of a combination of Haynes’ sensitive strategies for clinical prediction guides and etiology/risk, plus an added section related to prognostic factors and the statistical methods we are seeking.

Additional file 1 presents the complete search strategy. We restricted the search to adults, non-animal studies, and non-conference proceedings/abstracts from Embase but without date or language restrictions.

### Data abstraction

Data abstraction will be performed in duplicate by paired groups of reviewers. Standardized data collection forms will be created for reviewers to use when extracting data.

We will record the following data from each eligible article: author, year of publication, study type (retrospective, prospective, post hoc analysis of trial), name of trial if post hoc analysis of trial, whether single-center or multicenter, country of study, inclusion criteria, exclusion criteria, recruitment time frame (months), follow-up length (months), total sample size, definition of outcome (specific definition of mortality and timing of outcome), number of events, baseline demographics of cohort (e.g., sex, age, etiology of renal failure, body mass index) number of predictors included in the regression model, measure of point estimate of risk used (relative risk, odds ratio, hazard ratio), predictor, unit of change for continuous predictors, category for categorical predictors, measured point estimate of risk (odds ratio, hazard ratio), and lower and upper confidence interval. Kaplan-Meier survivor function will provide an estimated cumulative incidence of graft loss at 1-year post transplantation. If Kaplan-Meier curves are not available, data tables will provide the required data. All extracted data values will be rounded to the nearest integer.

### Data synthesis

We plan on generating point estimates and respective 95% confidence intervals (CI) using hazard ratios (HRs). We will convert all estimates from each individual study to HR using baseline risk estimates from that study. If baseline risk estimates are not reported and conversion is not possible, we will conduct subgroup analyses to compare studies based on the format of effect estimate (OR, RR, and HR). Specifically, we will compare studies presenting effect estimates using OR or RR, not reporting baseline risk (thus conversion not possible) with studies using HR together with studies reporting OR or RR but converted to HR due to the presence of baseline risk. If no significant difference is observed, given the low risk of 1-year mortality, we will pool all risk estimates and present as HR. In this subgroup analysis, we combined studies reporting in OR or RR together due to the low risk of mortality in the first year post transplant. We will pool the HR estimates for each predictor through inverse variance analysis using random-effects meta-analysis. In studies with stratified groups, if we observed a linear association between the predictor and outcome, we will average the beta coefficients across categories to obtain the estimate of effect associated with a unit change.

Certain publications may only report on significant predictors of mortality. Such studies raise problems of selective reporting bias as they do not report effect estimate and 95% confidence interval for variables fitted in the model, but not significantly associated with graft loss. For such studies, we will impute a relative effect estimate of 1 and utilize the hot deck approach for imputing the variance [6]. Subsequently, we will conduct a sensitivity analysis including the imputed studies.

We will consider a two-sided *p* value of 0.05 or less which indicates statistical significance. Review Manager 5 will provide the software for statistical analyses, as well as forest plots and funnel plots.

### Quality assessment (risk of bias within studies)

We will assess the study risk of bias using a modified version of the Quality in Prognostic Studies (QUIPS) instrument [7] (Additional file 3). We will assess the study risk of bias using six domains (study participation, study attrition, prognostic factors, outcome measurement, study confounding, statistical analysis and reporting). We modified the QUIPS tool to rate each domain as low or high risk of bias as opposed to the original low, medium, and high risk of bias. Under the statistical analysis and reporting domain, we modified the QUIPS to assess if the models are overfitted (less than 10 events for each binary prognostic factor). We will use the individual domains, rated as low, moderate, or high risk of bias, to inform the overall risk of bias in each study: five or six low-risk domains as overall low risk of bias, two or more high-risk domains as overall high risk of bias. Paired reviewers will independently assess each included study using the modified QUIPS tool.

### Sources of heterogeneity

We will address the statistical heterogeneity through consistency of point estimates and the extent of overlap of confidence interval. Heterogeneity will not be assessed with *I*^2^ statistics, as this is uniformly high and thus not useful in prognostic studies with very large sample size and precise estimates [8].

We will conduct subgroup analyses to explore heterogeneity across studies. We will focus on two possible effect modifiers: definition of mortality, duration of follow-up, and a number of aspects relating to the risk of bias. We will conduct the following a priori subgroup analyses to explain heterogeneity:

1. Definition of mortality (comparing studies reporting on all-cause mortality, composite of mortality, re-transplantation, and graft loss, or composite of re-transplantation and graft loss.
2. We will include studies assessing mortality beyond the first year only if Cox proportional hazard model is used to assess the independent impact of each prognostic factor. Therefore, we will conduct a subgroup analysis comparing the magnitude of effect estimate between studies specifically measuring mortality at 1 year to those looking at mortality beyond the first year. Certain prognostic factors may have a stronger association with graft loss at 1 year, and thus, the inclusion of follow-up time beyond the first year may attenuate the magnitude of effect estimate.
3. For each predictor, we will conduct a subgroup analysis to compare the effects in studies at high risk of bias with those at low risk of bias.

### Confidence in estimate of effect

For assessing the overall confidence in certainty evidence, we will use the Grading of Recommendations, Assessment, Development, and Evaluation (GRADE) approach considering the risk of bias, imprecision, inconsistency, indirectness and publication bias [8]. Confidence could be rated as high, moderate, low, or very low. Because we are addressing the issue of prognosis and not causation, observational studies will start as high confidence. We will assess publication bias using funnel plots and visual inspection of symmetry. The GRADE approach is typically applied at the outcome level. Since the focus of our review is prognostic factors for the same outcome, assessment of confidence in certainty of evidence will be applied at the level of each individual prognostic factor.

If the pooled estimate of our sensitivity analysis (including imputed non-significant studies evaluating the predictor of interest) differs from our primary pooled estimate on the same predictor (not including the imputed non-significant predictors predictor), we will attribute more credibility and apply the GRADE assessment to the sensitivity analysis and rate down for risk of bias.

## Discussion

One operational challenge with our proposed review is the applicability of the GRADE tool for evaluating certainty in the evidence. Specifically, when assessing certainty, we need to address the presence of imprecision. Assessment of imprecision will require absolute effect estimates. To obtain absolute effect estimate, we will require baseline risk estimates obtained from patients who do not have the prognostic factor under evaluation. Not all included studies will require baseline risk estimates of graft survival in patients without the prognostic factor under evaluation. For this, we may need to refer to indirect evidence from registry studies to assess imprecision. This is a foreseeable issue that may arise with the applicability of GRADE. The GRADE working group for prognosis is currently working on developing guidance for the application of their tool when assessing the certainty of evidence for individual prognostic factors. We will conduct this review coincident with the development of their guidance which may ease our assessment for the certainty of the evidence.

The results of this review may be beneficial for a number of future applications:

1. Transplant physicians and patients may be interested in an accurate assessment of their prognosis. This review may provide information that physicians and patients can use in conversations regarding the extent of benefit they can expect from lung transplantation. If there are multiple prognostic factors that modify the risk of 1-year mortality, physicians with limited time may be challenged with combining the factors together to identify the specific risk of their patient. For this reason, risk prediction models may serve as an attractive tool in discussions of prognosis. To date, however, the performance of the existing risk prediction models for 1-year mortality is just acceptable (AUC 0.67) [9]. Evidence from this review could inform the development of future risk prediction models that will result in improved prediction.
2. This review will identify and summarize all predictors evaluated by individual studies. Small studies have access to more granular specific variables that may prove to be predictors of 1-year mortality. Public access to a repository of such studies will be a useful starting point for authors of future reviews that may be interested in evaluating these factors when more studies are available.
3. Finally, the current opioid crisis [10], efforts in expanding the pool of donors [11], and an ever-growing interest in better understanding the management of deceased donors [12] may change the risk of 1-year mortality. For this reason, it is useful to better understand the risk of 1-year mortality by identifying and exploring the characteristics of the low- and high-risk lung transplant recipients.

## Additional files

Additional file 1: Search strategy. (DOCX 23 kb)

Additional file 2: Study eligibility form. (DOCX 18 kb)

Additional file 3: Risk of bias for predictor studies. (DOCX 17 kb)

## Data Availability

All supplementary data is made available.

## Abbreviations

GRADE: Grading of Recommendations Assessment, Development and Evaluation
HR: Hazard ratio
ISHLT: International Society of Heart and Lung Transplantation
OR: Odds ratio
QUIPS: Quality in Prognostic Studies
RR: Risk ratio
UNOS: United Network for Organ Sharing
USA: United States of America
