# Supplementary material for: Predictors of 1-year mortality in adult lung transplant recipients: a systematic review and meta-analysis
Source: Syst Rev. 2019 Jun 3;8:131. doi: 10.1186/s13643-019-1049-x (PMC6547526; doi:10.1186/s13643-019-1049-x)
Supplement: Supplementary file 1 — Search strategy. (DOCX 23 kb) [file 13643_2019_1049_MOESM1_ESM.docx]

**APPENDIX A – Search Strategy**

Database: Ovid MEDLINE(R) <1946 to March Week 3 2017>

Search Strategy:

--------------------------------------------------------------------------------

1 [lung transplantation] (0)

2 exp Lung Transplantation/ (13998)

3 exp lung/tr (105)

4 (lung? adj2 transplant*).mp. (17043)

5 (lung? adj2 graft*).mp. (579)

6 (lung? adj2 allograft*).mp. (1207)

7 (lung? adj2 allotransplant*).mp. (209)

8 (lung? adj2 heterograft*).mp. (0)

9 (lung? adj2 heterotransplant*).mp. (7)

10 (lung? adj2 homotransplant*).mp. (26)

11 (lung? adj2 homograft*).mp. (8)

12 (pulmonary adj2 transplant*).mp. (851)

13 (pulmonary adj2 graft*).mp. (287)

14 (pulmonary adj2 allograft*).mp. (289)

15 (pulmonary adj2 allotransplant*).mp. (15)

16 (pulmonary adj2 heterograft*).mp. (10)

17 (pulmonary adj2 heterotransplant*).mp. (2)

18 (pulmonary adj2 homotransplant*).mp. (13)

19 (pulmonary adj2 homograft*).mp. (383)

20 (cardiopulmonary adj2 transplant*).mp. (184)

21 (cardiopulmonary adj2 graft*).mp. (430)

22 (cardiopulmonary adj2 allograft*).mp. (3)

23 (cardiopulmonary adj2 allotransplant*).mp. (2)

24 (cardiopulmonary adj2 heterograft*).mp. (0)

25 (cardiopulmonary adj2 heterotransplant*).mp. (0)

26 (cardiopulmonary adj2 homotransplant*).mp. (2)

27 (cardiopulmonary adj2 homograft*).mp. (2)

28 or/2-27 (18727)

29 [graft loss etc] (0)

30 exp Host vs Graft Reaction/ (86666)

31 (graft? adj2 loss*).mp. (6005)

32 (transplant adj2 loss*).mp. (214)

33 Primary Graft Dysfunction/ (480)

34 (graft? adj2 dysfunction*).mp. (3006)

35 (transplant adj2 dysfunction*).mp. (406)

36 (graft? adj2 fail*).mp. (10103)

37 (transplant adj2 fail*).mp. (979)

38 (graft? adj2 survival*).mp. (49822)

39 (transplant adj2 survival*).mp. (2126)

40 exp mortality/ (330045)

41 mo.fs. (494027)

42 or/30-41 (759856)

43 28 and 42 (6711)

44 [predictors/risk/prognostic factors] (0)

45 predict*.mp. (1116690)

46 scor*.tw. (629282)

47 observ*.mp. (2638814)

48 validat*.mp. (353136)

49 exp risk/ (1007713)

50 risk*.mp. (1892186)

51 exp Cohort Studies/ (1650322)

52 between group*.tw. (85712)

53 exp prognosis/ (1352527)

54 (prognos* adj2 factor*).mp. (75370)

55 (prognos* adj2 value*).mp. (36679)

56 (associat* adj2 factor*).mp. (121225)

57 (independent adj2 factor*).mp. (58971)

58 (multivariate adj2 factor*).mp. (3166)

59 (multivariable* adj2 factor*).mp. (513)

60 exp Regression Analysis/ (360244)

61 regression*.mp. (549354)

62 (hazard* adj2 model*).mp. (76125)

63 (cox adj2 model*).mp. (14721)

64 (hazard* adj2 ratio*).mp. (67230)

65 exp survival analysis/ (236746)

66 or/45-65 (7000435)

67 43 and 66 (4447)

68 animals/ not (animals/ and humans/) (4329339)

69 67 not 68 (4160)

70 limit 69 to ("all infant (birth to 23 months)" or "all child (0 to 18 years)") (1178)

71 limit 69 to "all adult (19 plus years)" (2935)

72 69 not 70 (2982)

73 71 or 72 (3863)

74 remove duplicates from 73 (3781)

Database: Ovid MEDLINE(R) Epub Ahead of Print and In-Process & Other Non-Indexed Citations <March 23, 2017>

Search Strategy:

--------------------------------------------------------------------------------

1 (lung? adj2 transplant*).mp. (1298)

2 (lung? adj2 graft*).mp. (43)

3 (lung? adj2 allograft*).mp. (116)

4 (lung? adj2 allotransplant*).mp. (2)

5 (lung? adj2 heterograft*).mp. (0)

6 (lung? adj2 heterotransplant*).mp. (0)

7 (lung? adj2 homotransplant*).mp. (0)

8 (lung? adj2 homograft*).mp. (0)

9 (pulmonary adj2 transplant*).mp. (63)

10 (pulmonary adj2 graft*).mp. (33)

11 (pulmonary adj2 allograft*).mp. (13)

12 (pulmonary adj2 allotransplant*).mp. (0)

13 (pulmonary adj2 heterograft*).mp. (0)

14 (pulmonary adj2 heterotransplant*).mp. (0)

15 (pulmonary adj2 homotransplant*).mp. (0)

16 (pulmonary adj2 homograft*).mp. (20)

17 (cardiopulmonary adj2 transplant*).mp. (14)

18 (cardiopulmonary adj2 graft*).mp. (22)

19 (cardiopulmonary adj2 allograft*).mp. (0)

20 (cardiopulmonary adj2 allotransplant*).mp. (1)

21 (cardiopulmonary adj2 heterograft*).mp. (0)

22 (cardiopulmonary adj2 heterotransplant*).mp. (0)

23 (cardiopulmonary adj2 homotransplant*).mp. (0)

24 (cardiopulmonary adj2 homograft*).mp. (0)

25 or/1-24 (1429)

26 (graft? adj2 loss*).mp. (645)

27 (transplant adj2 loss*).mp. (22)

28 (graft? adj2 dysfunction*).mp. (319)

29 (transplant adj2 dysfunction*).mp. (25)

30 (graft? adj2 fail*).mp. (1004)

31 (transplant adj2 fail*).mp. (109)

32 (graft? adj2 survival*).mp. (1414)

33 (transplant adj2 survival*).mp. (306)

34 mortal*.mp. (74315)

35 or/26-34 (77014)

36 25 and 35 (388)

Database: Embase <1974 to 2017 March 23>

Search Strategy:

--------------------------------------------------------------------------------

1 exp lung transplantation/ (31300)

2 (lung? adj2 transplant*).mp. (36600)

3 (lung? adj2 graft*).mp. (2621)

4 (lung? adj2 allograft*).mp. (2092)

5 (lung? adj2 allotransplant*).mp. (258)

6 (lung? adj2 heterograft*).mp. (0)

7 (lung? adj2 heterotransplant*).mp. (9)

8 (lung? adj2 homotransplant*).mp. (23)

9 (lung? adj2 homograft*).mp. (8)

10 (pulmonary adj2 transplant*).mp. (1369)

11 (pulmonary adj2 graft*).mp. (462)

12 (pulmonary adj2 allograft*).mp. (385)

13 (pulmonary adj2 allotransplant*).mp. (20)

14 (pulmonary adj2 heterograft*).mp. (10)

15 (pulmonary adj2 heterotransplant*).mp. (1)

16 (pulmonary adj2 homotransplant*).mp. (12)

17 (pulmonary adj2 homograft*).mp. (564)

18 (cardiopulmonary adj2 transplant*).mp. (263)

19 (cardiopulmonary adj2 graft*).mp. (528)

20 (cardiopulmonary adj2 allograft*).mp. (3)

21 (cardiopulmonary adj2 allotransplant*).mp. (3)

22 (cardiopulmonary adj2 heterograft*).mp. (0)

23 (cardiopulmonary adj2 heterotransplant*).mp. (0)

24 (cardiopulmonary adj2 homotransplant*).mp. (2)

25 (cardiopulmonary adj2 homograft*).mp. (3)

26 or/1-25 (39025)

27 graft dysfunction/ (5238)

Annotation: Did not explode but chose the relevant narrower terms.

28 delayed graft function/ (4711)

29 graft failure/ (31128)

30 primary graft dysfunction/ (1211)

31 lung graft rejection/ (1741)

32 (graft? adj2 loss*).mp. (12152)

33 (transplant adj2 loss*).mp. (425)

34 (graft? adj2 dysfunction*).mp. (9442)

35 (transplant adj2 dysfunction*).mp. (655)

36 (graft? adj2 fail*).mp. (36385)

37 (transplant adj2 fail*).mp. (1873)

38 (graft? adj2 survival*).mp. (63703)

39 (transplant adj2 survival*).mp. (4652)

40 exp mortality/ (971440)

41 or/27-40 (1062643)

42 26 and 41 (11870)

43 predict*.tw. (1588973)

44 exp methodology/ (5081887)

45 validat*.tw. (525071)

46 risk*.mp. (3065038)

47 exp epidemiology/ (2941671)

48 prognosis/ (597088)

49 prognostic assessment/ (3274)

50 (prognos* adj2 factor*).mp. (125881)

51 (prognos* adj2 value*).mp. (61042)

52 (associat* adj2 factor*).mp. (189556)

53 (independent adj2 factor*).mp. (98764)

54 (multivariate adj2 factor*).mp. (5304)

55 (multivariable* adj2 factor*).mp. (956)

56 exp regression analysis/ (541847)

57 regression*.mp. (854778)

58 (hazard* adj2 model*).mp. (126317)

59 (cox adj2 model*).mp. (29455)

60 (hazard* adj2 ratio*).mp. (115817)

61 survival analysis/ (3722)

62 (survival adj2 analy*).mp. (56984)

63 or/43-62 (9944135)

64 42 and 63 (9606)

65 (exp animals/ or exp animal experimentation/ or nonhuman/) not ((exp animals/ or exp animal experimentation/ or nonhuman/) and exp human/) (5969916)

66 64 not 65 (9362)

67 limit 66 to (embryo <first trimester> or infant <to one year> or child <unspecified age> or preschool child <1 to 6 years> or school child <7 to 12 years> or adolescent <13 to 17 years>) (1338)

68 limit 66 to (adult <18 to 64 years> or aged <65+ years>) (3671)

69 66 not 67 (8024)

70 68 or 69 (8824)

71 limit 70 to (book or book series or chapter or conference abstract or conference paper or conference proceeding or "conference review") (3729)

72 70 not 71 (5095)

73 remove duplicates from 72 (4820)

Database: EBM Reviews - Cochrane Database of Systematic Reviews <2005 to March 22, 2017>

Search Strategy:

--------------------------------------------------------------------------------

1 (lung? adj2 transplant*).mp. (52)

2 (lung? adj2 graft*).mp. (4)

3 (lung? adj2 allograft*).mp. (1)

4 (lung? adj2 allotransplant*).mp. (0)

5 (lung? adj2 heterograft*).mp. (0)

6 (lung? adj2 heterotransplant*).mp. (0)

7 (lung? adj2 homotransplant*).mp. (0)

8 (lung? adj2 homograft*).mp. (0)

9 (pulmonary adj2 transplant*).mp. (2)

10 (pulmonary adj2 graft*).mp. (1)

11 (pulmonary adj2 allograft*).mp. (0)

12 (pulmonary adj2 allotransplant*).mp. (0)

13 (pulmonary adj2 heterograft*).mp. (0)

14 (pulmonary adj2 heterotransplant*).mp. (0)

15 (pulmonary adj2 homotransplant*).mp. (0)

16 (pulmonary adj2 homograft*).mp. (0)

17 (cardiopulmonary adj2 transplant*).mp. (0)

18 (cardiopulmonary adj2 graft*).mp. (1)

19 (cardiopulmonary adj2 allograft*).mp. (0)

20 (cardiopulmonary adj2 allotransplant*).mp. (0)

21 (cardiopulmonary adj2 heterograft*).mp. (0)

22 (cardiopulmonary adj2 heterotransplant*).mp. (0)

23 (cardiopulmonary adj2 homotransplant*).mp. (0)

24 (cardiopulmonary adj2 homograft*).mp. (0)

25 or/1-24 (56)

26 (graft? adj2 loss*).mp. (62)

27 (transplant adj2 loss*).mp. (6)

28 (graft? adj2 dysfunction*).mp. (7)

29 (transplant adj2 dysfunction*).mp. (1)

30 (graft? adj2 fail*).mp. (79)

31 (transplant adj2 fail*).mp. (13)

32 (graft? adj2 survival*).mp. (61)

33 (transplant adj2 survival*).mp. (18)

34 mortal*.mp. (4689)

35 or/26-34 (4724)

36 25 and 35 (47)

Database: EBM Reviews - Cochrane Central Register of Controlled Trials <February 2017>

Search Strategy:

--------------------------------------------------------------------------------

1 exp Lung Transplantation/ (186)

2 exp lung/tr (0)

3 (lung? adj2 transplant*).mp. (601)

4 (lung? adj2 graft*).mp. (66)

5 (lung? adj2 allograft*).mp. (27)

6 (lung? adj2 allotransplant*).mp. (0)

7 (lung? adj2 heterograft*).mp. (0)

8 (lung? adj2 heterotransplant*).mp. (0)

9 (lung? adj2 homotransplant*).mp. (0)

10 (lung? adj2 homograft*).mp. (0)

11 (pulmonary adj2 transplant*).mp. (23)

12 (pulmonary adj2 graft*).mp. (14)

13 (pulmonary adj2 allograft*).mp. (1)

14 (pulmonary adj2 allotransplant*).mp. (0)

15 (pulmonary adj2 heterograft*).mp. (0)

16 (pulmonary adj2 heterotransplant*).mp. (0)

17 (pulmonary adj2 homotransplant*).mp. (0)

18 (pulmonary adj2 homograft*).mp. (8)

19 (cardiopulmonary adj2 transplant*).mp. (4)

20 (cardiopulmonary adj2 graft*).mp. (191)

21 (cardiopulmonary adj2 allograft*).mp. (1)

22 (cardiopulmonary adj2 allotransplant*).mp. (0)

23 (cardiopulmonary adj2 heterograft*).mp. (0)

24 (cardiopulmonary adj2 heterotransplant*).mp. (0)

25 (cardiopulmonary adj2 homotransplant*).mp. (0)

26 (cardiopulmonary adj2 homograft*).mp. (0)

27 or/1-26 (850)

28 exp Host vs Graft Reaction/ (2758)

29 (graft? adj2 loss*).mp. (809)

30 (transplant adj2 loss*).mp. (39)

31 Primary Graft Dysfunction/ (13)

32 (graft? adj2 dysfunction*).mp. (218)

33 (transplant adj2 dysfunction*).mp. (36)

34 (graft? adj2 fail*).mp. (1027)

35 (transplant adj2 fail*).mp. (72)

36 (graft? adj2 survival*).mp. (2641)

37 (transplant adj2 survival*).mp. (222)

38 exp mortality/ (10980)

39 mo.fs. (21900)

40 or/28-39 (29512)

41 27 and 40 (193)

PubMed supplemental for non-Medline records

| **Query** | **Items found** |
| --- | --- |
| **Search** (((((((("lung"[All Fields] OR "lungs"[All Fields] OR "pulmonary"[All Fields] OR "cardiopulmonary"[All Fields])) AND ("transplant"[All Fields] OR "transplants"[All Fields] OR "transplantation"[All Fields] OR "transplanted"[All Fields] OR "graft"[All Fields] OR "grafts"[All Fields] OR allograft*[All Fields] OR allotransplant*[All Fields] OR heterograft*[All Fields] OR heterotransplant*[All Fields] OR homotransplant*[All Fields] OR homograft*[All Fields]))) AND ((((("graft"[All Fields] OR "grafts"[All Fields] OR "transplant"[All Fields])) AND (loss*[All Fields] OR dysfunction*[All Fields] OR fail*[All Fields] OR survival*[All Fields]))) OR mortal*[All Fields]))) AND ((((((((((((predict*[Title/Abstract] OR scor*[Title/Abstract] OR observ*[Title/Abstract] OR "validation"[Title/Abstract] OR "validate"[Title/Abstract] OR risk*[Title/Abstract] OR "cohort"[Title/Abstract])) OR group*[Text Word]) OR regression*[Text Word]) OR (hazard*[Text Word] AND model*[Text Word])) OR ("cox"[Text Word] AND model*[Text Word])) OR (hazard*[Text Word] AND ratio*[Text Word]))) OR (((prognos*[Text Word] OR associat*[Text Word] OR "independent"[Text Word] OR "multivariate"[Text Word] OR multivariable*[Text Word])) AND (factor*[Text Word] OR variable*[Text Word]))))) OR (survival[Text Word] AND analy*[Text Word])))) AND (pubstatusaheadofprint OR publisher[sb] OR pubmednotmedline[sb]) | 586 |
