# Supplementary material for: Predictors of 1-year mortality in adult lung transplant recipients: a systematic review and meta-analysis
Source: Syst Rev. 2019 Jun 3;8:131. doi: 10.1186/s13643-019-1049-x (PMC6547526; doi:10.1186/s13643-019-1049-x)
Supplement: Supplementary file 2 — Study eligibility form. (DOCX 18 kb) [file 13643_2019_1049_MOESM2_ESM.docx]

**APPENDIX B – STUDY ELIGIBILITY FORM**^1^

| **Population:** | | |
| --- | --- | --- |
| - Lung Transplant Recipients | **YES** | **NO** |
| - Adults (≥ 18 years old) | **YES** | **NO** |

| **Predictor:** | | |
| --- | --- | --- |
| - Any predictor of mortality (see outcome details below) | **YES** | **NO** |

| **Adjusted Analysis:** | | |
| --- | --- | --- |
| - Multivariate analysis | **YES** | **NO** |

| **Outcomes reported:** | | |
| --- | --- | --- |
| - Mortality within the first year post transplant OR Graft loss at anytime post transplant ONLY IF authors used Cox regression analysis (Hazard Ratio, HR) | **YES** | **NO** |

| **Type of article:** | | |
| --- | --- | --- |
| - Cohort study (retrospective or prospective) or | **YES** | **NO** |
| - RCT cohort (post-hoc analysis) |  |  |

| **Duplicated population:** |  |  |
| --- | --- | --- |
| - If duplicated, does this study provide new information? | **YES** | **NO** |
| - If duplicated, is study more recent? |  |  |

| **Study inclusion:** |  |
| --- | --- |
| - All the answers are YES | **INCLUDE** |
| - Any answer is NO | **EXCLUDE** |
| - If you are unsure of the answer, include for full text screening | **INCLUDE** |

**Instructions:**

^1^ On occasion, some of the above criteria, especially during T&A screening, will be unclear. If any response to the above questions is UNCLEAR, mark YES.

^2^ Consider YES if any type of predictor, including but not limited to clinical characteristics, laboratory values, test results and any other clinical event

^3^Exclude studies evaluating therapies as a predictor and not reporting on any other potential predictor.

^5^ Consider NO if the study used only any other type of adjustment for potential confounders, including matched design or stratification.

^6^ Outcomes could be analyzed at any time point during follow up if hazard ratio is reported. 1-year mortality should be present for studies using odds ratio.

^7^ Consider YES if it is a post-hoc analysis of an RCT evaluating other predictors and not just a therapy. Meta-analysis on observational studies evaluating a therapy could be included in the individual studies used multivariable analysis. We will abstract information related to predictors from individual studies if they fulfill the rest of this project inclusion criteria.
