# Supplementary material for: Predictors of 1-year mortality in adult lung transplant recipients: a systematic review and meta-analysis
Source: Syst Rev. 2019 Jun 3;8:131. doi: 10.1186/s13643-019-1049-x (PMC6547526; doi:10.1186/s13643-019-1049-x)
Supplement: Supplementary file 3 — Risk of bias for predictor studies. (DOCX 17 kb) [file 13643_2019_1049_MOESM3_ESM.docx]

**APPENDIX C - Risk of Bias for Predictor Studies**

**Modified version of QUIPS**

1. Study Participation. Adequately described:

- Source of target population.

- Sampling frame and recruitment method.

- Period of recruitment.

- Place of recruitment.

- Inclusion/exclusion criteria

- Baseline key characteristics

2. Study Attrition. Adequately described:

- Lost to follow-up.

- Attempts at collecting information on patients lost to follow-up.

- Reasons for lost to follow-up.

- Key characteristics of participants lost to follow-up.

- No differences between patients who completed and those lost to follow-up

3. Prognostic Factors. Adequately described:

- Definition of prognostic factors (if definition required).

- Methods of prognostic factor measurement (if measurement required).

- Adequate proportion of the study sample has complete data on prognostic factor.

- Methods of imputation for dealing with missing prognostic factor data.

4. Outcome. Adequately described:

- Definition of outcome (including duration of follow-up).

- Methods of outcome measurement (if measurement required).

- Methods and setting of outcome measurement is the same for all study participants.

5. Study Confounding. Adequately described:

- All important confounders, including treatments.

- Important confounders are accounted for in the final regression model.

6. Statistical Analysis and Reporting. Adequately described:

- Analytic strategy.

- Model building (low risk of bias for building model based on conceptual framework.

- No overfitting of final model (1 variable for every 10 events).

- Checking of model assumptions.

- Reporting on all variables included in the final model (statistically significant or not).
